# Medical Items

**Published:** 1915-07

**Authors:** 


### MEDICAL ITEMS

### THE HAY FEVER PROBLEM.

This is the time of year when the services of the physician are actively demanded by the victim of vasmotor rhinitis—a season dreaded not alone by the patient, but, not uncommonly, by his medical adviser as well. Particularly is this true of the latter if he has not kept abreast of modern ideas on the therapy of hay fever. In any event the disease is one that tries the patience and calls for the application of remedial agents that have been proved beyond peradventure. Happily there are a number of such agents from which the physician can choose—products that have passed the experimental stage and demonstrated their serviceability. We refer in this connection to some members of the Adrenalin family—Adrenalin Chloride Solution, Adrenalin Inhalent, Anesthone Cream, Anesthone Inhalent. These products, in all of which the isolated active principle of the suprarenal gland (Adrenalin) is an active constituent, have rendered long, efficient service in the treatment of hay fever, and one feels no hesitancy in heartily commending them.

Adrenalin Chloride Solution, which is perhaps more widely used than any other preparation in the treatment of hay fever, is sprayed into the nasal chambers and pharynx by means of a hand atomizer adapted for aqueous liquids, or it may be applied on a pledget of cotton. For the former purpose it is advisable to dilute the solution as marketed (1:1000) by the addition of four to five times its volume of physiologic salt solution.

Adrenalin Inhalent, which is a solution, is an aromatized neutral oil base, of the suprarenal actice principle, is well adapted for vaporization and inhalation from an oil atomizer. Used as an adjunct to Adrenalin Chloride Solution, or independently, it gives good results, parts not accessible to other medication being readily reached by the medicated vapor. It should be diluted by the addition of three to four times its volume of olive oil.

